# A Preliminary Study of microRNA-208b after Acute Myocardial Infarction: Impact on 6-Month Survival

**DOI:** 10.1155/2018/2410451

**Published:** 2018-05-27

**Authors:** Mostafa Alavi-Moghaddam, Mohammad Chehrazi, Shamila D. Alipoor, Maryam Mohammadi, Alireza Baratloo, Mohammad Parsa Mahjoub, Mehrnaz Movasaghi, Johan Garssen, Ian M. Adcock, Esmaeil Mortaz

**Affiliations:** ^1^Emergency Medicine Department, Imam Hossein Hospital, Shahid Beheshti University of Medical Sciences, Tehran, Iran; ^2^Clinical Immunology Department, National Research Institute of Tuberculosis and Lung Diseases (NRITLD), Shahid Beheshti University of Medical Sciences, Tehran, Iran; ^3^Department of Epidemiology and Reproductive Health, Reproductive Epidemiology Research Center, Royan Institute for Reproductive Biomedicine, ACECR, Tehran, Iran; ^4^Institute of Medical Biotechnology, Molecular Medicine Department, National Institute of Genetic Engineering and Biotechnology (NIGEB), Tehran, Iran; ^5^Emergency Medicine Department, Tehran University of Medical Sciences, Tehran, Iran; ^6^Department of Cardiology, Imam Hossein Hospital, Shahid Beheshti University of Medical Sciences, Tehran, Iran; ^7^Clinical Tuberculosis and Epidemiology Research Center, National Research Institute for Tuberculosis and Lung Disease (NRITLD), Shahid Beheshti University of Medical Sciences, Tehran, Iran; ^8^Division of Pharmacology, Faculty of Science, Utrecht Institute for Pharmaceutical Sciences, Utrecht University, Utrecht, Netherlands; ^9^Nutricia Research Centre for Specialized Nutrition, Utrecht, Netherlands; ^10^Cell and Molecular Biology Group, Airways Disease Section, Faculty of Medicine, National Heart and Lung Institute, Imperial College London, London, UK; ^11^Priority Research Centre for Asthma and Respiratory Disease, Hunter Medical Research Institute, University of Newcastle, Newcastle, NSW, Australia; ^12^Department of Immunology, School of Medicine, Shahid Beheshti University of Medical Sciences, Tehran, Iran

## Abstract

**Introduction:**

miRNAs contribute to a variety of essential biological processes including development, proliferation, differentiation, and apoptosis. Circulating microRNAs are very stable and have shown potential as biomarkers of cardiovascular disease. microRNA-208b expression was increased in the blood of patients with acute myocardial infarction (AMI) and has been proposed as a biomarker for early diagnosis. In this pilot study, we investigate the potential of circulating miR-208b as a prognostic biomarker of 6-month survival in AMI patients.

**Methods:**

Plasma samples from 21 patients and 8 age- and gender-matched healthy adults were collected, and circulating levels of miR-208b were detected using quantitative real-time PCR.

**Results:**

miR-208b levels were higher in healthy control subjects (9.6-fold; *P* ≤ 0.05). Within the AMI patients, the levels of miR-208b were significantly lower in the *survivor* versus *nonsurvivor* group (fold change = 6.51 and 14.1, resp.; *P* ≤ 0.05). The Kaplan-Meier curve revealed that the 6-month survival time was significantly higher among AMI patients with a relative expression of miR-208b lower than 12.38. The hazard ratio (HR) for the relative expression of miR-208b (<12.38 was the reference) was 5.08 (95% CI: 1.13–22.82; *P* = 0.03).

**Conclusion:**

Our results showed that elevated miR-208b expression was associated with reduced long-term survival in AMI patients. These pilot data indicate the need for a large follow-up study to confirm whether miR-208b can be used as a predictor of 6-month survival time after AMI.

## 1. Introduction

Acute myocardial infarction (AMI) occurs as a result of the acute necrosis of myocardial tissue following persistent and severe ischemia [1]. AMI is one of the most common cardiovascular diseases and one of the leading causes of mortality and morbidity across the globe. 17 million people die annually of cardiovascular diseases with 10 million being in developing countries [2–4]. Patients with a comorbid diagnosis of AMI had two to three times the case-fatality rate of patients in whom AMI was a primary diagnosis [5]. It is predicted that cardiovascular diseases will constitute 36% of all deaths globally in 2020 [6]. Some conventional biomarkers, such as blood troponins, cardiac myoglobin, and creatine kinase-MB (CK-MB) are currently used for clinical diagnosis of AMI [7].

microRNAs (miRNAs) are small (19–25 nucleotides in length), noncoding, and highly conserved RNA molecules which are involved in the regulation of gene expression. The regulatory functions of miRNAs are achieved through the RNA-induced silencing complex [8]. miRNAs control a variety of essential biological processes including development, proliferation, differentiation, and apoptosis [9]. Dysregulated tissue expression of miRNAs contributes to various diseases such as cancer and cardiovascular disease [10–12]. Recent studies demonstrated that miRNAs play a crucial role in AMI mechanisms such as atherosclerotic plaque rupture, blood platelet aggregation, and necrosis of heart cells after blockage of the coronary artery [13].

Many miRNAs are remarkably stable and easily detectable in the peripheral blood or plasma [14, 15]. The levels of circulating miRNAs may differ under pathological conditions [16–18]. This suggests plasma miRNA concentrations may be used as superior biomarkers for the diagnosis and prognosis of diseases in humans [19, 20]. The levels of several miRNAs such as miR-1, miR-133a, miR-208b, miR-499, and miR-328 are altered in the blood and plasma during AMI [21–26]. miR-149, miR-499, and miR-208b are increased immediately after percutaneous coronary intervention (PCI) and therefore have promise as diagnostic and prognostic biomarkers in AMI [27]. miR-208 is produced exclusively in the rat myocardium and is considered as a biomarker of myocardial injury in rats [28]. The same study reported that the plasma level of miR-499 may also be a useful biomarker of myocardial infarction in humans [28]. The present study aimed to investigate the potential prognostic value of circulating miR-208b in AMI patients with respect to 6-month survival time.

## 2. Methods

### 2.1. Patient Characteristics

This pilot prospective prognostic study recruited AMI patients sequentially referred to the Imam Hossein Hospital affiliated to the Shahid Beheshti University of Medical Sciences between January and December 2016. The study was approved by the Research Ethics Committee of Shahid Beheshti University of Medical Sciences at Tehran, Iran, and all patients gave informed consent.

Patients with acute ischemic chest pain, abnormal electrocardiogram (pathological Q wave and ST-segment elevation), and increased levels of troponin and creatine kinase greater than 2 times the upper limit of the normal range with a diagnosis of AMI were enrolled into the study. Patients with a previous history of venous thrombolytic injection or receiving anticoagulant, previous MI or PCI, hematological diseases, acute or chronic infection, significant hepatic dysfunction, renal failure, or known or cured malignancy were excluded. The patients were admitted to hospital no more than 12 h after the emergence of symptoms, and blood samples were collected immediately after admission. A cutoff value of 55% was used for the ejection fraction (EF). If the diagnosis of AMI was confirmed, then the blood samples were submitted to the reference laboratory for miRNA analysis.

Five-milliliter venous blood samples of patients with AMI were collected in EDTA anticoagulant tubes at admission. Samples were centrifuged at 3000 ×g for 10 min at 4°C, and then the supernatant was isolated and centrifuged at 12,000 ×g for 10 min at 4°C. Plasma was collected and stored at −80°C until RNA extraction. Moreover, 8 age- and gender-matched healthy volunteers with normal electrocardiograms and no history of cardiovascular diseases were recruited as a control group.

### 2.2. RNA Extraction and cDNA Synthesis

Serum-free miRNAs in patients and the control group were extracted using an RNA extraction kit (Exiqon, Vedbæk, Denmark). Extracted RNA was reverse transcribed using the miRCURY LNA Universal RT microRNA cDNA Synthesis Kit (Exiqon) according to the manufacturer's instructions.

### 2.3. Real-Time Quantitative PCR Analysis

#### 2.3.1. Quantitative Reverse Transcription-Polymerase Chain Reaction (qRT-PCR)

Real-time PCR assays were performed using the ExiLENT SYBR® Green Master Mix Kit (Exiqon). LNA primers were purchased from Exiqon. cDNA was diluted 10x and added to the PCR reactions according to the manufacturer's instructions. The real-time PCR program included the following steps: an initial denaturation step at 95°C for 10 min and 50 cycles of amplification that consisted of a denaturation step (10 s at 95°C) and an annealing step (60 s at 60°C). The expression levels of miR-208b were normalized to the level of miR-16 as control using the efficiency-corrected calculation models of the Pfaffle method [29].

(1) $$\begin{array}{c} Ratio=\frac{\left(Etarget\right)\;\Delta Ct\;target\left(control-sample\right)}{\left(ERef\right)\;\Delta Ct\;Ref\left(control-sample\right)}. \end{array}$$

### 2.4. Statistical Analysis

Data were presented using mean (SD) and frequency and 95% confidence interval (95% CI). Independent sample *t*-tests or Mann–Whitney *U* tests and chi-square test were used to investigate the differences in continuous and categorical variables, respectively. The Kaplan-Meier method was used for depicting univariate survival curves illustrating the association between the biomarker expression and disease-specific survival (DSS). DSS was defined from the date of enrollment until the time of AMI death. Statistical significance between the survival curves was assessed utilizing the log-rank test. The Cox proportional hazard model was used to estimate the hazard ratio of death for miR-208b. The cutoff value was determined based on the Youden index. *P* values less than 0.05 were considered statistically significant for all analyses. All statistical analyses were performed using the statistical package IBM SPSS, version 21 (SPSS Inc., Chicago, IL, USA).

## 3. Results

### 3.1. Clinical Characteristics of Patients

Among the 21 patients diagnosed with AMI, 7 patients died within six months of diagnosis. Both the nonsurvivor and the survivor groups were predominantly male (6/7 and 9/14, resp.). The demographics of the patients in this study are shown in Table 1. No significant differences were observed in the personal history including hypertension, hyperlipidemia, diabetes, cardiac troponin T, and the left ventricular ejection fraction (EF) and smoking between nonsurvivor and survivor patients.

### 3.2. Assessment of the Circulating miR-208b Levels

**Figure 1:**
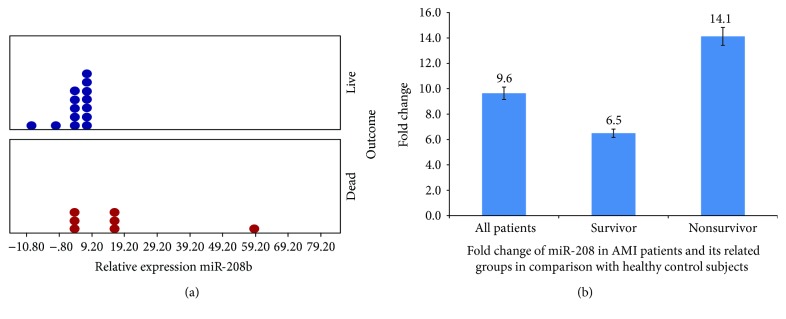
(a) Relative expression of circulating miR-208b in AMI patients in the survivor and nonsurvivor groups (*P* = 0.03). The expression of miR-208b was 9.6-fold higher in the AMI group compared with the healthy control subjects. (b) The relative expression of miR-208b was increased in both survivor and nonsurvivor groups in comparison to healthy subjects (fold change = 6.51 and 14.1, resp.). Plasma samples were collected upon admission no more than 24 h after AMI onset (in all cases, *P* value ≤ 0.05).

The level of miR-208b was measured in the plasma of 21 AMI patients according to the survival time as well as in the healthy controls. The expression of miR-208b was significantly greater in the AMI group compared with healthy control subjects (fold change = 9.6, *P* ≤ 0.05) (Figure 1). As shown in Figure 1, the relative expression of miR-208b was increased in both survivor and nonsurvivor groups as compared to healthy subjects (fold change = 6.51 and 14.1, resp.; *P* ≤ 0.05). There was no effect of age on the miR-208b level (Spearman *r* = 0.2049 and *P* value (two-tailed) = 0.4148).

The result also showed a significant difference in the plasma level of miR-208b between the survivor and nonsurvivor groups in AMI patients (Figure 1). The plasma level of circulating miR-208b in nonsurvivors was 2.1-fold higher than that in survivors (Figure 1).

### 3.3. Survival Analysis

**Figure 2:**
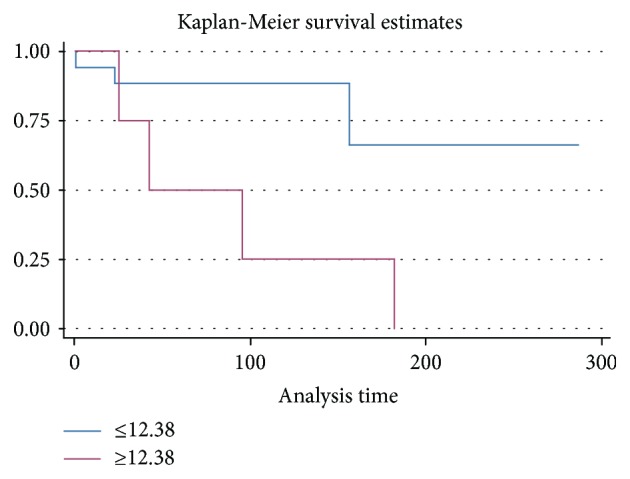
Kaplan-Meier curve displaying the survival in relation to high or low miR-208b relative expression.

The influence of the clinical characteristics on the median and 6-month survival is presented in Table 2. Only the relative expression of miR-208b (*P* = 0.02) was a significant prognosticator.  Table 3 presents the results from the Cox regression analyses regarding the clinical variables and their impact on the survival. All the variables showed no significant relation with survival in univariate analyses except the relative expression of miR-208b. To compare survival function according to levels of miR-208b, we put a cutoff point of 12.38 which was obtained by using the Youden index. High relative expression of miR-208b was the most significant negative prognostic factor in our patient cohort (HR: 5.08; 95% CI: 1.13–22.82; *P* = 0.03) (Figure 2).

## 4. Discussion

In this study, we investigated the prognostic value of miR-208b to predict the 6-month survival time for patients with ST-elevation myocardial infarction (STEMI). qRT-PCR analysis confirmed that baseline plasma levels of miR-208b were greater in AMI patients compared with healthy controls. An important finding in this study was that the levels of miR-208b on admission had a significant ability to predict 6-month survival time. The survival curves indicated that a relative expression cutoff of 12.38 for circulating miR-208b clearly distinguished the survival odds.

The diagnosis and treatment of AMI patients is of paramount importance, and predictive factors could have widespread application in clinical practice [2]. Cardiac troponins and creatine kinase-MB are the most common biomarkers for AMI diagnosis. However, their detection may be limited in some cases. Thus, measuring the levels of circulating miRNAs might provide an additional specific biomarker for the diagnosis and treatment of AMI. In addition, higher plasma levels of miR-208b have also been significantly associated with the risk of death during a 6-month follow-up in acute coronary syndrome (ACS) [30]. Thus, a combination of plasma miR-208b detection with clinical characterization may give greater prognosis of 6-month survival in patients with different types of heart disease.

*miR*-*208b* is encoded by intron 31 within the MYH7 gene and regulates the expression of its host gene via the Sox6 transcription factor. The MYH7 gene encodes the beta- (*β*-) myosin heavy chain 7 that is found in the heart (cardiac) muscle [31]. MiR-208b is considered a cardiac-specific miRNA with an important role in human heart function and cardiopathology [32]. During the early stages of AMI, this miRNA might leak out of the necrotic myocardium and be released into the circulation [33]. Cardiomyocyte-enriched miRNAs have been recently considered as the potential diagnostic biomarkers in AMI due to their rapid release, cardiac selectivity, and plasma stability [30]. Previous studies reported that the plasma level of some miRNAs such as miR-1, miR-133a, miR-133b, miR-208a, and miR-499 was significantly elevated in patients with STEMI compared to healthy controls and patients with chest pain but normal coronary angiograms [32–34].

Plasma levels of miR-208b have been previously studied in relation to their predictive value in AMI [35]. Plasma miR-208b levels were higher in AMI patients, and ROC analysis gave AUC values of 0.72. An even greater predictive ROC value (0.94) for miR-208b in AMI was reported by Corsten and colleagues [33]. Several other studies have reported elevated miR-208b levels in AMI compared with healthy control subjects which have implied a role for miR-208b as a diagnostic marker [36–38]. However, none of these studies considered the rate of survival post-AMI.

A rapid increase in the level of circulating miR-208b after myocardial infarction is correlated with decreased systolic function, increased rejection fraction, and increased expression of markers of cardiomyocyte necrosis [39]. It is plausible that following myocardial damage, cardio-enriched miRNAs such as miR-208b are released into the bloodstream from necrotic cardiomyocytes which subsequently have a paracrine effect on the heart [40]. For example, miR-208b may exacerbate the deleterious conditions within the myocardium post-MI and increase the risk of death or development of heart failure [41]. As such, strategies designed to inhibit miR-208b may be of therapeutic value. In support of this concept, inhibition of miR-208a improves cardiac function and survival during heart failure [31] in addition to acting as a potential noninvasive biomarker of myocardial injury [31].

The effect of age and age-related diseases such as cancer and cardiovascular disease on the expression of circulating miRNAs has been previously examined [42]. An age-dependent decrease in miRNA expression in peripheral blood mononuclear cells (PBMCs) was seen along with lower serum levels of miR-151a-5p, miR-181a-5p, and miR-1248 [42–44]. Our data failed to show a clear effect of age on miR-208b expression, but further studies using subjects with a greater age range should be investigated.

## 5. Conclusion

Our result confirms previous studies demonstrating a possible role of miR-208b as a candidate biomarker for AMI diagnosis [27] and extends this to show that the precise level of miR-208b in these patients on admission was a good indicator of 6-month survival. Thus, the relative expression of miR-208b was significantly increased in AMI patients who died within 6 months compared to those AMI patients who survived, but larger studies are required to confirm this.

### 5.1. Limitations

There were some limitations to our study. Our findings are based on a small sample size, and further research with a larger sample size with a longer follow-up time is required to obtain accurate and reproducible results. Repeated measurements of miR-208b levels at more time points would also enhance the reliability of our findings.

## Figures and Tables

**Table 1:** Clinical features and risk factors of the cohort.

Characteristics	All (*n* = 21)	Survivor (*n* = 14)	Nonsurvivor (*n* = 7)	*P* value
Age	62.71 (12.75)	58.57 (11.37)	71 (11.88)	0.15
Sex (M/F)	15/6	9/5	6/1	0.31
Smoking (yes/no)	8/13	5/9	3/4	0.75
Diabetes (yes/no)	4/17	3/11	1/6	0.69
Hypertension (yes/no)	13/8	8/6	5/2	0.52
Hyperlipidemia (yes/no)	10/11	6/8	4/3	0.54
Cardiac troponin T (ng/mL)	10.08 (10.18)	10.54 (10.20)	9.15 (10.89)	0.77
Decrease in EF^∗^ (yes/no)	6/13	4/10	2/3	0.64
Relative expression	8.63 (12.83)	4.58 (4.85)	16.72 (19.58)	0.04

^∗^Cutoff = 0.55.

**Table 2:** Clinical variables as predictors of the survival all AMI patients and differentiated into lower and upper 12.38 subgroups (*N* = 21, 17, and 4, resp., univariate analyses; log-rank test) in 21 AMI patients.

Characteristics	Patient *N* (NOE)	Median survival (days)	6-month survival (%)	*P* value
*Relative expression*				
<12.38	17 (3)	NR	66.2	**0.02**
≥12.38	4 (4)	43	0	
*Gender*				
Female	6 (1)	182	75	0.32
Male	15 (6)	NR	36.7	
*Smoking*				
Yes	8 (3)	182	47	0.81
No	13 (4)	157	37.5	
*Diabetes*				
Yes	4 (1)	NR	75	0.69
No	17 (6)	182	34.3	
*Hypertension*				
Yes	13 (5)	182	42.7	0.75
No	8 (2)	157	43.8	
*Hyperlipidemia*				
Yes	10 (4)	NR	52.5	0.32
No	11 (3)	182	45.5	
*Decrease in EF * ^∗^				
Yes	6 (2)	182	41.7	0.85
No	13 (3)	NR	51.3	

NOE: number of events; NR: not reached. ^∗^Cutoff = 0.55.

**Table 3:** Results of Cox regression analyses for clinical variables and miR-208 relative expression among AMI patients.

Characteristics	HR	95% CI	*P* value
*Age*	**1.04**	**(0.98–1.10)**	**0.15**
*Cardiac troponin T (ng/mL)*	**0.97**	**(0.87–1.05)**	**0.33**
*Relative expression*			
<12.38	Reference		**0.03**
≥12.38	5.08	(1.13–22.82)	
*Gender*			
Female	Reference		0.35
Male	2.81	(0.33–23.91)	
*Smoking*			
No	Reference		0.81
Yes	1.21	(0.27–5.48)	
*Diabetes*			
No	Reference		0.69
Yes	0.66	(0.08–5.48)	
*Hypertension*			
No	Reference		0.75
Yes	1.30	(0.25–6.77)	
*Hyperlipidemia*			
No	Reference		0.33
Yes	2.16	(0.46–10.13)	
*Decrease in EF * ^∗^			
No	Reference		0.85
Yes	1.19	(0.19–7.53)	

HR: hazard ratio. ^∗^Cutoff = 0.55.

## Data Availability

The data used to support the findings of this study are available from the corresponding author upon request.
